# Differential Effects of Maternal Yolk Androgens on Male and Female Offspring: A Role for Sex-Specific Selection?

**DOI:** 10.1371/journal.pone.0133673

**Published:** 2015-07-20

**Authors:** Barbara Tschirren

**Affiliations:** Institute of Evolutionary Biology and Environmental Studies, University of Zurich, Zurich, Switzerland; Universidad de Granada, SPAIN

## Abstract

Maternal hormones are important mediators of prenatal maternal effects in animals. Although their effects on offspring phenotype are often sex-specific, the reason why sometimes sons are more sensitive to prenatal hormone exposure and sometimes daughters is not well understood. Here I combine an experimental manipulation of yolk testosterone concentration in the egg and quantification of selection acting on yolk androgen-sensitive traits in a natural population of great tits (*Parus major*) with a literature review to test the hypothesis that sex-specific selection on traits affected by yolk androgens determines which sex is more sensitive to prenatal hormone exposure. An experimental increase of the testosterone content in the egg boosted the post-hatching growth of male, but not female great tit nestlings. However, I found no evidence that survival selection on body mass or size is acting differently in the two sexes. A literature review revealed that yolk androgen manipulations affect the growth of males and females differently across species. Interestingly, in studies performed in the wild a significant association between the strength and direction of sexual size dimorphism and sex-specific sensitivities to yolk androgens was observed. In studies performed in captivity, no such relationship was found. Thus, across species there is some evidence that sex-specific selection on body size influences how strongly growth trajectories of males and females are affected by maternally-derived yolk androgens.

## Introduction

Environmental conditions encountered early in life can modify developmental processes and can have long-term effects on the morphology, physiology and behavior of organisms [1]. Mothers can considerably influence the environment encountered by the offspring during early development by differentially transferring resources and cues to the developing young [1]. Important mediators of such prenatal maternal effects are hormones that are transferred by the mother during pregnancy [2,3] or egg laying [4]. Experimental studies have demonstrated that maternal hormones, and in particular maternal androgens, can affect various aspects of offspring phenotype, including growth, physiology and behavior (reviewed in [5,6]). Interestingly, these effects are often sex-specific or even sexually antagonistic. However, the reason why sometimes sons are more sensitive to maternal hormones and sometimes daughters is not well understood [5].

In birds, male sex is the default and females differentiate because of the actions of estrogen [7]. Thus, one would not necessarily expect that males benefit more strongly from high yolk androgen exposure than females. Because testosterone can be aromatized into estrogen, high yolk testosterone concentrations may even be detrimental for males [7,8]. Thus, on a proximate level it is difficult to predict which sex should benefit from maternal androgen transfer.

Instead, an ultimate perspective might help to understand why yolk androgens have sex-specific effects. It has been proposed that traits under sex-specific selection may be particularly susceptible to non-genetic modifiers of development [9]. If selection acts in sex-specific ways on traits affected by yolk androgens, this could explain different yolk androgen sensitivities in the two sexes. Here I test this hypothesis by combining a yolk androgen manipulation experiment with a quantification of sex-specific selection acting on traits (i.e. fledging mass and fledging size) that are potentially affected in a sex-specific way by yolk androgens in a natural great tit (*Parus major*) population. Furthermore, I review previously published studies that tested for sex-specific consequences of yolk androgens on growth, size and mass in other bird species, and associate sex-specific effects of yolk androgens on growth within species with the strength and direction of sexual size dimorphism, which is the outcome of sex-specific natural and sexual selection, across species. If sex differences in the strength of selection acting on yolk androgen-sensitive traits influence how strongly the phenotype of males and females is modulated by yolk androgens, I predict that the growth of females is more strongly boosted in response to a yolk androgen manipulation in species where females are the larger sex, and vice versa.

## Materials and Methods

### Yolk hormone manipulation

The yolk hormone manipulation was performed in a nestbox-breeding population of great tits (*Parus major*) in Switzerland (46°54’N 7°17’E / 46°57’N 7°21’E). Nestboxes (Typ Varia, 12.5cm x 12.5cm x 26.5 cm) were regularly checked to determine the start of nest building and egg laying. One day after clutch completion, I manipulated the yolk androgen content in the eggs (N = 268) as described in [10]. In short, I injected all eggs of a clutch with either 12 ng testosterone (T)(17β-hydroxy-4-androsten-3-on) (Fluka, Switzerland) dissolved in 5 μl of sesame oil (T nests: N = 13 clutches) or 5 μl of sesame oil as a control (C nests: N = 18 clutches). Clutches were randomly assigned to treatment groups by tossing a coin. Injections were performed on a cold light (Volpi, Switzerland) using a Hamilton syringe and a 25G needle. The hole in the shell was sealed with a drop of tissue adhesive (Vetseal, B Braun Medical, Switzerland). Because of the left-skewed distribution of yolk testosterone concentrations across clutches, the injected dose ensured that > 95% of the eggs had a yolk testosterone content that was, although experimentally increased, within the natural range of yolk testosterone contents observed in great tits (mean ± 1SD: 8.8 ± 3.7 ng / yolk, maximum: 26.4 ng / yolk) [11].

### Nestling measures

On day 1 post-hatching, nestlings were marked individually by clipping down feathers and they were weighed (thereafter: hatching mass; N = 85 nestlings from a testosterone-injected egg; N = 99 control nestlings). On day 8 post-hatching, nestlings were marked with a numbered aluminum ring and a small amount of blood (approx. 20 μl) was taken from the metatarsal vein and stored in 98% ethanol for molecular sex determination (N = 46 females from a testosterone-injected egg; N = 35 males from a testosterone-injected egg; N = 54 control females; N = 37 control males). A tissue sample was collected if a nestling was found dead in the nest. I measured nestling body mass and tarsus length, a proxy for body size in birds, on day 15 post-hatching, shortly before fledging (thereafter: fledging mass and size; N = 42 females from a testosterone-injected egg; N = 26 males from a testosterone-injected egg; N = 33 control females; N = 23 control males).

### Ethical statement

All non-hunted bird species are protected under Swiss laws. The study did not involve endangered species. All procedures comply with the current laws of Switzerland and were approved by the Animal Experimentation Board of the Cantonal Veterinary Office of Berne, Berne, Switzerland (license 16/02) and the Federal Office for the Environment, Berne, Switzerland (license 2200). The municipality of Berne approved field work in their forests.

### Molecular sexing

Nestlings were sexed following the protocol of [12]. In short, DNA was extracted using a commercial kit (DNeasy Blood & Tissue Kit, Qiagen) following the manufacturer’s protocol. PCR was carried out in a total volume of 10μl using the primers P2 / P8 as described in [13]. PCR products were separated with electrophoresis at 80 V for 30 min on a 2% agarose gel stained with SYBR Safe DNA Gel Stain (Invitrogen). The sex of 23 nestlings could not be determined because they disappeared from the nest (i.e. died and were removed by the parents) before a blood or tissue sample could be collected.

### Statistical analysis of hormone manipulation experiment

Hatching mass and fledging mass and size were analysed with general linear mixed models that included hormone manipulation, nestling sex and their two-way interaction as fixed effects. Nest ID, nested in hormone manipulation, was included as a random effect to account for the non-independence of nestlings growing up in the same nest. Brood size and hatching date were included as covariates to account for variation in nestling measures due to sibling competition and seasonal variation in food availability. Residuals of the models were tested for normality and homoscedasticity. Least squares means contrasts were used to test for treatment effects within sexes.

For the analysis of hatching success, survival and sex ratio, I used generalised linear mixed models with a binomial error structure using the glmer function, part of the package lme4 [14]. The same random and fixed effects were included as for the general linear mixed models.

### Sex-specific selection on fledging size and mass

To test if selection acts in a sex-specific way on traits that are differentially affected by yolk testosterone in males and females, I analysed if fledging size and / or mass are differentially associated with local recruitment in males and females. Recruits were identified when feeding their brood, based on the unique ring number they had received as a nestling. Local recruitment was analysed using generalised linear mixed models with a binomial error structure [14]. Nest ID was included as a random effect, and sex, phenotype (fledging size or mass), and their interaction were included as fixed effects. Data on 467 nestlings (45 were recorded as breeders during the following year) from 86 non-experimental broods from the same study population and year were included in this analysis.

To obtain formal estimates of the direction and strength of selection acting on fledging size and mass in males and females, I standardized fledging size and mass to have a mean of zero and a standard deviation of one, and calculated relative fitness for each bird by dividing recruitment by the population mean recruitment rate. I then fitted linear regression models that included standardized fledging size or fledging mass for the estimation of standardized directional selection gradients (*β*), and quadratic regression models that included standardized fledging size or fledging mass and their quadratic term for the estimation of quadratic selection gradients (*γ*) [15]. Quadratic regression coefficients were doubled to obtain estimates of *γ* [16].

### Sex-specific effects of yolk hormones in other bird species

To examine sex-specific consequences of maternal yolk androgens on growth in other bird species, I performed a systematic literature search in Web of Science using the search terms ‘yolk testosterone’, ‘yolk androgen’ or ‘yolk hormone’, and ‘offspring growth’, ‘manipulation and offspring’, ‘sex difference’ or ‘sex-specific’. I subsequently checked the references in these papers for relevant studies that were missed with this search. Only studies that experimentally manipulated yolk androgen concentrations in bird eggs and measured sex-specific effects on growth and morphology in the offspring were considered.

I determined for each study if an experimental increase of yolk androgen levels was more beneficial (in terms of enhanced growth) for males, females or if the treatment effect was similar in the two sexes. Furthermore, for each species in the dataset, I determined sexual dimorphism in body mass by calculating Lovich & Gibbons’ sexual dimorphism index (SDI; body mass larger sex / body mass smaller sex—1) [17]. The resulting value is arbitrarily made negative if the male is the larger sex and positive if the female is the larger sex [17]. I obtained data on average male and female body mass from the authors (Table 1) or, if not available, from [18]. I used body mass rather than tarsus length to determine SDI because tarsus lengths of males and females were not available for all species. Finally, I determined if a study was performed in the wild or in captivity as the consequences of yolk androgens might be context-dependent.

**Table 1:** Sex-specific effects of yolk androgen manipulation on growth and morphology in birds.

Species	Body mass (g)	SDI	Manipulated hormone	Wild / Captive	Trait	Age	Effect in Males	Effect in Females	Comments	References
American kestrel *Falco sparverius*	Female: 141.3 Male: 97.8 (A)	0.445	T +A4	Wild	Growth rate / Body size	juv	M –	F =		[20]
Barn swallow *Hirundo rustica*	Female: 19.0 Male: 17.8 (A)	0.067	T + A4	Wild	Growth	juv	M +	F –	No significant difference in fledging mass or size	[21]
Canary *Serinus canaria*	Female: 20.2 Male: 19.0	0.063	T	Captive	Growth	juv	M +	F +		[22]
Canary *Serinus canaria*	Female: 20.2 Male: 19.0 (A)	0.063	T	Captive	Growth / Beak width	juv	M–/ –	F + / +	Complex interactions with age and hatching asynchrony	[23,24,25]
Collared flycatcher *Ficedula albicollis*	Female: 15.5 Male: 13.0 (A)	0.192	T + A4	Wild	Tarsus length / Local recruitment	juv / ad	M–/ –	F + / =	Significant interaction effect, but non-significant within-sex contrasts	[26,27,28]
Collared flycatcher *Ficedula albicollis*	Female: 15.4 Male: 13.1 (A)	0.176	T + A4	Wild	Fledging mass	juv	M =	F =		[29]
Chicken Gallus gallus domesticus	Female: 1786.0 Male: 2365.0 (A)	-0.324	A4	Captive	Tarsus length	juv	M =	F +		[30]
Great tit *Parus major*	Female: 16.8 Male: 17.1 (A)	-0.018	T	Wild	Fledging mass / Tarsus length	juv	M +	F =		This study
House sparrow *Passer domesticus*	Female: 27.4 Male: 28.0 (D)	-0.022	T	Captive	Body mass / Survival	ad	M +	F + / ++		[31]
Pied flycatcher *Ficedula hypoleuca*	Female: 12.7 Male: 12.2 (A)	0.041	T + A4	Wild	Tarsus length	juv	M –	F –		[32]
Spotless starling *Sturnus unicolor*	Female: 81.0 Male: 88.05 (A)	-0.087	T + A4; T; A4; T & A4	Wild	Growth / Body condition	juv	M + / –	F + / –		[33,34]
Yellow-legged gull *Larus michahellis*	Female: 1033.0 Male: 1275.0 (D)	-0.234	T	Wild	Body mass	juv	M –	F –		[35]
Zebra finch *Taeniopygia guttata*	Female: 12.2 Male: 11.9 (D)	0.025	T	Captive	Growth / Body condition	juv	M–/ –	F + /––	Complex interactions with age, no difference in fledging mass	[36,37]

Body mass: A: data from authors; D: data from Dunning (2008).

SDI: sexual dimorphism index.

Manipulated hormone: T: testosterone; A4: androstenedione.

Effects: + increased trait value; ++ strongly increased trait value;–reduced trait value;––strongly reduced trait value; = trait not affected.

I then tested the hypothesis that the strength and direction of SDI in a species predicts if an experimental increase of yolk androgen levels boosts the growth of females more strongly, of males more strongly, or if both sexes show a similar response to the manipulation by means of a multinomial regression analysis. The response to the yolk androgen manipulation (females benefit, males benefit, both sexes react similarly) was included as the dependent variable and SDI, study type (wild / captive) and their interaction was included as explanatory variables. Each study population was entered only once in the analysis. Analyses were performed in R 2.14.2 [19] or JMP 10.0 (SAS Institute Inc., Cary, NC, USA). Tests were two-tailed with a significance level set at P ≤ 0.05.

## Results

### Effects of yolk hormone manipulation on hatching success and hatching mass

Overall hatching success was 74%. Testosterone manipulated eggs (84%) tended to have a higher hatching success than control eggs (66%), but the difference did not reach statistical significance (*χ* ^*2*^ = 3.259, *DF* = 1, *P* = 0.071). There was no seasonal effect on hatching success (*χ* ^*2*^ = 0.836, *DF* = 1, *P* = 0.361).

There was no significant difference in hatching mass between nestlings originating from a testosterone manipulated (mean ± SD, 2.10 ± 0.60g, N = 85) or a control egg (mean ± SD, 1.93 ± 0.48g, N = 99)(*F*_1, 22.79_ = 0.462, *P* = 0.503), no significant difference in hatching mass between male (mean ± SD, 2.06 ± 0.64g, N = 67) and female nestlings (mean ± SD, 2.04 ± 0.44g, N = 96)(*F*_1, 141.6_ = 0.427, *P* = 0.514), and no significant interaction effect between sex and hormone manipulation on hatching mass (*F*_1, 142.5_ = 0.047, *P* = 0.829). There was no difference in brood size between testosterone-manipulated and control broods (mean ± SD, testosterone-manipulated: 8.3 ± 1.3, N = 13; control: 8.9 ± 1.5, N = 18) (*F*_1, 29_ = 1.444, *P* = 0.239).

### Sex-specific effects of yolk hormone manipulation on fledging size and mass

**Fig 1:**
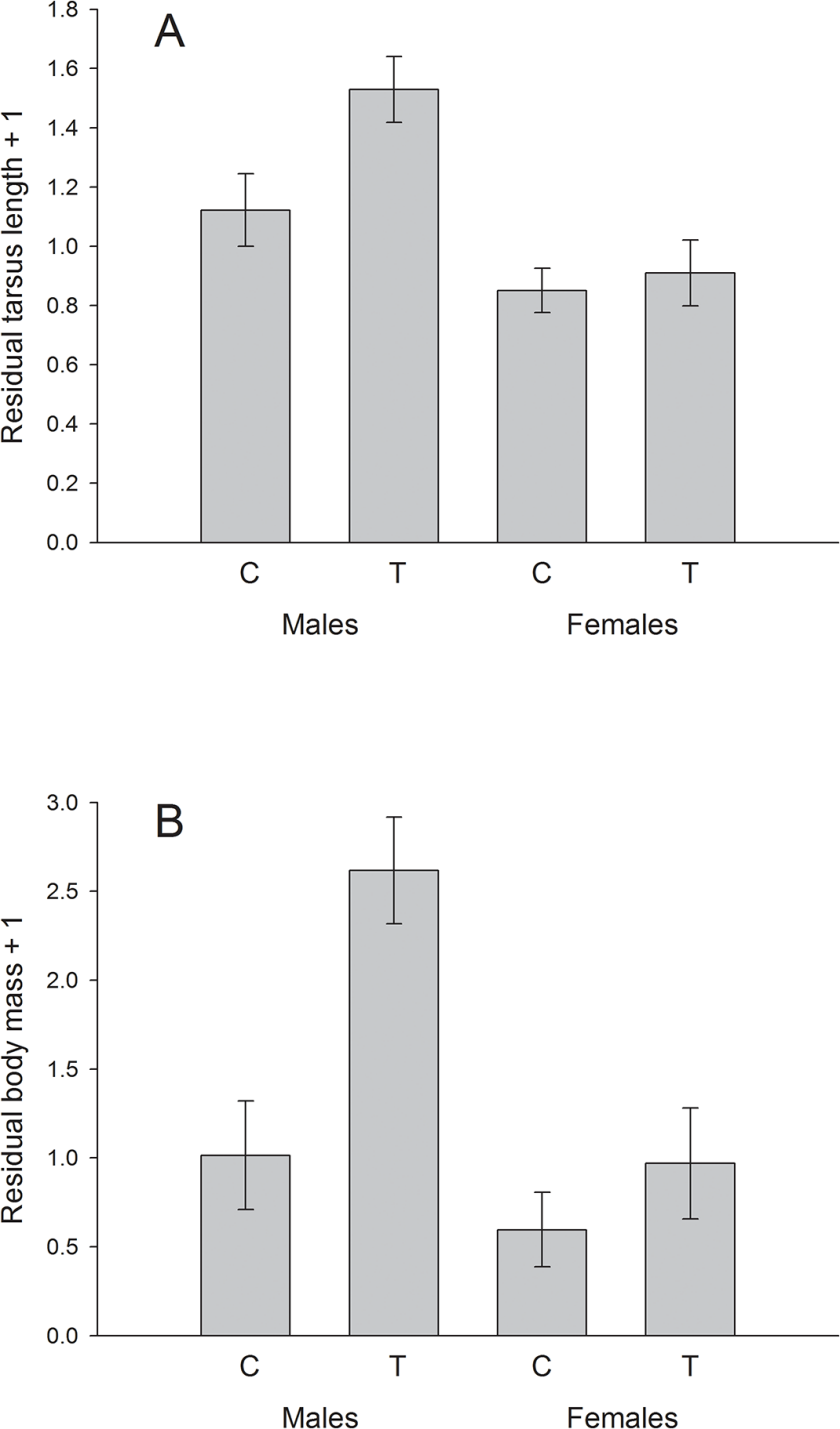
Sex-specific effects of yolk androgens in great tits. Difference in A) fledging size and B) fledging mass of male and female great tit nestlings originating from an egg with experimentally increased yolk testosterone content (T) or a control egg (C). Means ± 1 SE of the residuals (+1) of an analysis that included nest ID, brood size and hatching date are shown. Size difference between C and T males: *P* = 0.021, size difference between C and T females: *P* = 0.477; mass difference between C and T males: *P* = 0.026, mass difference between C and T females: *P* = 0.136.

Shortly before fledging, male great tit nestlings were significantly larger (*F*_1, 106.6_ = 30.542, *P* < 0.001) and heavier (*F*_1, 101.9_ = 30.103, *P* < 0.001) than females. Furthermore, nestlings that developed in an egg with an experimentally increased yolk testosterone content tended to be larger (*F*_1, 20.38_ = 2.983, *P* = 0.099) and heavier (*F*_1, 20.39_ = 4.142, *P* = 0.055) than nestlings originating from a control egg. However, this treatment effect was sex-specific (interaction sex x hormone manipulation: fledging size: *F*_1, 106.8_ = 6.445, *P* = 0.013; Fig 1A; fledging mass: *F*_1, 102_ = 3.031, *P* = 0.085; Fig 1B). Males originating from a testosterone-manipulated egg (mean ± SD, 19.39 ± 0.59 mm, N = 35) were significantly larger than males originating from a control egg (mean ± SD, 19.07 ± 0.58 mm, N = 38) (*F*_1, 29.23_ = 5.979, *P* = 0.021), whereas no such difference was observed in female nestlings (T females: mean ± SD, 18.77 ± 0.76 mm, N = 46; C females: mean ± SD, 18.79 ± 0.42 mm, N = 54) (*F*_1, 23.51_ = 0.541, *P* = 0.477; Fig 1A). Similarly, males originating from a testosterone-manipulated egg (mean ± SD, 16.86 ± 1.54g, N = 35) were significantly heavier than males originating from a control egg (mean ± SD, 16.04 ± 1.94g, N = 38)(*F*_1, 24.74_ = 5.628, *P* = 0.026), whereas, again, no such difference was found in females (T females: mean ± SD, 15.24 ± 2.40g, N = 46; C females: mean ± SD, 15.52 ± 1.58g, N = 54)(*F*_1, 21.79_ = 2.396, *P* = 0.136; Fig 1B).

### Effect of yolk hormone manipulation on nestling survival and sex ratio

Survival from hatching until fledging was lower in larger broods (*χ* ^*2*^ = 3.848, *DF* = 1, *P* = 0.050), but it was not significantly different between male (67%) and female nestlings (75%) (*χ* ^*2*^ = 0.765, *DF* = 1, *P* = 0.382). Nestlings originating from an egg with experimentally increased testosterone content tended to survive better from hatching to fledging (84%) than controls (61%)(*χ* ^*2*^ = 2.973, *DF* = 1, *P* = 0.085). This treatment effect on survival was similar for male and female nestlings (female C: 61%, female T: 91%, male C: 61%, male T: 74%; sex x hormone manipulation: *χ* ^*2*^ = 1.322, *DF* = 1, *P* = 0.250). There was no seasonal effect (*χ* ^*2*^ = 0.600, *DF* = 1, *P* = 0.439), no brood size x sex (*χ* ^*2*^ = 2.379, DF = 1, P = 0.123) and no brood size x treatment interaction (*χ* ^*2*^ = 1.022, DF = 1, P = 0.312) on nestling survival. Furthermore, no difference in the sex ratio of testosterone-manipulated and control broods was observed (*χ* ^*2*^ = 0.207, *DF* = 1, *P* = 0.649).

### Sex-specific selection on fledging size and mass

Heavier fledglings were significantly more likely to recruit into the local breeding population (*χ* ^*2*^ = 6.527, *DF* = 1, *P* = 0.011). Importantly, this association between fledging mass and recruitment was similar in males (= 0.495) and females (= 0.383) (interaction fledging mass x sex: *χ* ^*2*^ = 0.010, *DF* = 1, *P* = 0.920). Fledging size was not significantly associated with local recruitment (*χ* ^*2*^ = 1.195, *DF* = 1, *P* = 0.274). Importantly, there was again no indication that selection is acting in different ways on fledging size in males (= 0.161) and females (= 0.225) (interaction fledging size x sex: *χ* ^*2*^ = 0.058, *DF* = 1, *P* = 0.809). Non-linear selection on fledgling mass (*χ* ^*2*^ = 0.740, *DF* = 1, *P* = 0.390; males: *γ* = -0.020; females: *γ* = 0.076) and fledgling size (*χ* ^*2*^ = 2.830, *DF* = 1, *P* = 0.093; males: *γ* = -0.084; females: *γ* = 0.006) was statistically non-significant.

### Sex-specific effects of yolk hormone manipulation in other bird species

**Fig 2:**
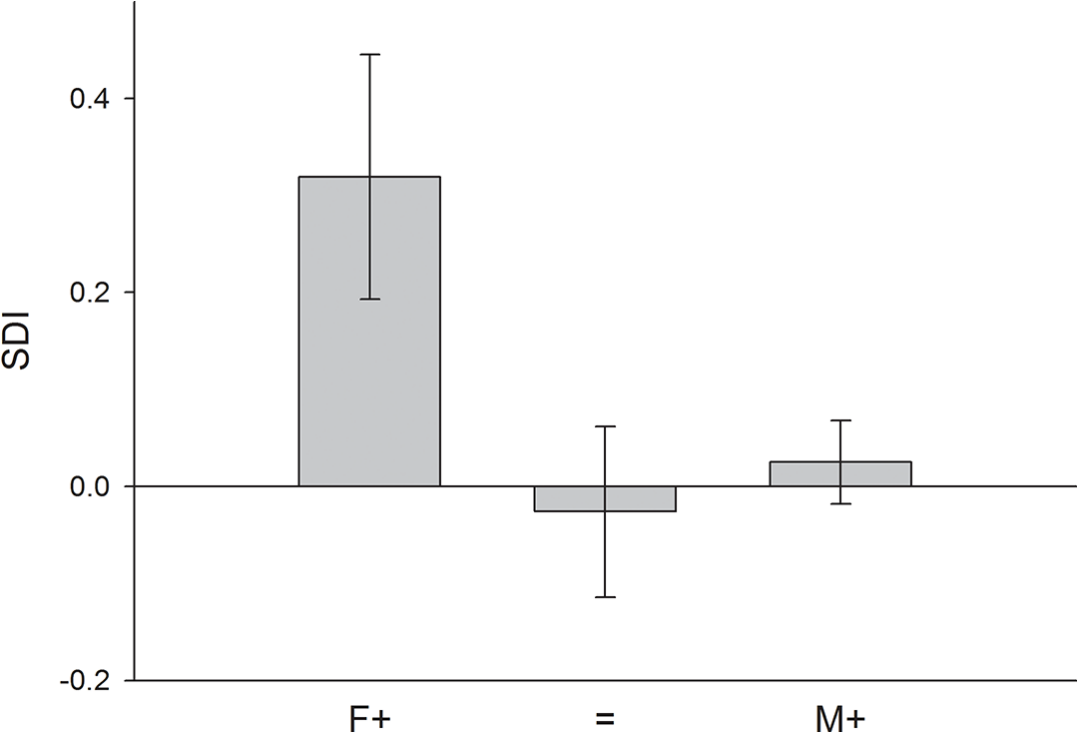
Differences in sexual size dimorphism between species that show sex-specific responses to yolk androgens in the wild. F+: species in which the growth of females was more strongly boosted by high yolk androgen levels; = species in which males and females showed a similar response to increased yolk androgen levels; M+: species in which the growth of males was more strongly boosted by high yolk androgen levels. SDI: sexual dimorphism index (body mass larger sex / body mass smaller sex—1). SDI is arbitrarily made negative if the male is the larger sex and positive if the female is the larger sex [17]. Means ± 1 SE of studies performed in the wild are presented. Note that differences in SDI between response groups are shown for illustrative purposes only. The statistical analyses were performed with response group as the dependent and SDI as the independent variable.

In total I found 18 published articles that experimentally tested for sex-specific consequences of yolk androgen manipulation on growth, mass and / or size of male and female birds. A summary of the findings of the different studies is presented in Table 1. A list of other traits (especially behavioral and physiological traits) that were found to be affected in a sex-specific way by yolk androgens is presented in S1 Table. Sex-specific effects of yolk androgen manipulation on growth or morphology were reported repeatedly. Sexually antagonistic effects (i.e. having significantly positive effects in one sex but significantly negative effects in the other), however, were comparably rare (four studies; Table 1). There was considerable variation among studies in which sex is more sensitive to maternal yolk hormones (Table 1). A multinomial regression analysis revealed that the interaction between SDI and study type (wild / captivity) explained a significant amount of variation in these sex-specific effects of yolk androgens (*χ* ^*2*^ = 8.534, *P* = 0.014; *N* = 13). When analyzing studies performed in the wild and in captivity separately, I found that in the wild SDI was a significant predictor of which sex benefits from exposure to high yolk androgen concentrations (*χ* ^*2*^ = 9.212, *P* = 0.010; *N* = 8; Fig 2), whereas in studies performed in captivity, no significant relationship between SDI and sex-specific responses to yolk androgen manipulation was observed (*χ* ^*2*^ = 1.654, *P* = 0.198; *N* = 5).

## Discussion

An experimental increase of the testosterone content in great tit eggs boosted the growth of male nestlings, whereas the growth of female nestlings was unaffected. A review of previously published studies that experimentally tested for sex-specific consequences of yolk androgens on growth, mass or size in other bird species revealed that this finding is not universal. Indeed, there appears to be no consistent pattern in which sex is more sensitive to yolk androgen exposure in terms of growth (Table 1, and references therein).

What causes these contrasting, sex-specific sensitivities to yolk androgens across species? It has been proposed that sex-specific selection on traits affected by yolk androgens might play a role [9]. To test this hypothesis, I used two different approaches. First, I tested if natural selection acts in a sex-specific way on fledging mass and size, which are differentially affected by yolk androgens in male and female great tit nestlings. To this end, I quantified the strength and direction of linear and non-linear selection acting on fledging mass and size via local recruitment in the two sexes. I found that heavier nestlings were more likely to recruit into the local breeding population the following year. Importantly, however, the strength and direction of selection was similar in males (*β* = 0.495) and females (*β* = 0.383). Also, there was no indication that selection on fledging size differed between the sexes (males: *β* = 0.161; females: *β* = 0.225). Thus, there was no evidence that survival selection is acting in a sex-specific way on two traits that were differentially affected by yolk androgens in male and female great tits.

However, it should be noted that I only assessed sex-specific selection on body mass and size in terms of local recruitment in this study and cannot exclude the possibility that other forms of selection, especially sexual selection, may act in a sex-specific way on mass and size in great tits (e.g. [38]). After all, males are larger and heavier than females in this species, and this difference is most likely the result of differential selection pressures acting on size and mass in the two sexes.

Second, I complied data on sexual size dimorphism and sex-specific effects of yolk androgen manipulations on growth and body size in other bird species to test the hypothesis that the sex which benefits more strongly from large body size is more sensitive to yolk androgen manipulation in terms of enhanced growth. To this end, I determined the strength and direction of sexual size dimorphism (SDI), which is the consequences of past sex-specific selection acting on male and female body size in a species, and tested if SDI predicts which sex modifies its growth trajectory more strongly in response to elevated yolk androgen concentrations across species. Interestingly, there was a significant interaction effect between SDI and study type (wild / captive) on sex-specific yolk androgen sensitivities. When analyzing wild and captive studies separately, a significant relationship between SDI and sex-specific yolk androgen sensitivities was observed in the wild; but no relationship was found in captive studies. The different patterns observed in the wild and in captivity suggest that the (sex-specific) effects of yolk androgens may be context-dependent, and influenced, for example, by food availability. However, I have to stress that sample sizes for the across species comparisons are very small and the lack of a relationship in captive studies might simply be due to low statistical power. Thus, studies in more species, both in the wild and in captivity, should be conducted to confirm the robustness of these results.

## Conclusions

In conclusion, I found mixed evidence for the hypothesis that differences in the strength of selection acting on mass and size in males and females predicts which sex modifies its developmental trajectories more strongly in response to yolk androgen exposure.

In the great tit experiment, I show that maternally-derived yolk testosterone has sex-specific effects on post-hatching growth. But no evidence was found that natural selection via local recruitment acts in a sex-specific way on these traits. However, given that males are larger and heavier than females, it is likely that other types of selection, especially sexual selection, may act in a sex-specific way on body size and mass in this species.

The across species analysis provided some evidence that the strength and direction of sexual size dimorphism across species predicts sex-specific sensitivities to yolk androgens within species. This association suggests that (sex-specific) sensitivities to non-genetic modifiers of development are moulded by natural selection.

## Supporting Information

S1 Table Behavioural and physiological traits that show sex-specific responses to yolk androgens in birds. (PDF)

S2 Table ARRIVE Checklist (PDF)
