# Supplementary material for: Differential Effects of Maternal Yolk Androgens on Male and Female Offspring: A Role for Sex-Specific Selection?
Source: PLoS One. 2015 Jul 20;10(7):e0133673. doi: 10.1371/journal.pone.0133673 (PMC4508120; doi:10.1371/journal.pone.0133673)
Supplement: S1 Table — (PDF) [file pone.0133673.s001.pdf]

Supplementary material Tschirren (2015) Differential Effects of Maternal Yolk Androgens on Male and Female Offspring: a Role for Sex-Specific Selection?

S1 Table. Behavioural and physiological traits that show sex-specific responses to yolk androgen manipulation in birds

Species	Manipulated hormone	Wild / Captive	Trait	Age	Effect in Males / Females	Reference
Black-headed gull<br>*Larus ridibundus*	T & A4	Wild	Immune response	juv	M –<br>F –	[1]
Chicken<br>*Gallus gallus*	T	Captive	Plasma T	juv	M –<br>F – (?)	[2]
Chicken<br>*Gallus gallus*	T	Captive	Androgen receptor expression in brain	juv	M –<br>F –	[2]
Eurasian Jackdaw<br>*Corvus monedula*	T & A4	Wild	Humoral & cell-mediated immune response	juv	M –<br>F –	[3]
House sparrow<br>*Passer domesticus*	T	Captive	Plumage colour / Latency to approach food source	ad	M + / –<br>F = / –	[4]
House sparrow<br>*Passer domesticus*	T	Captive	Antagonistic approaches towards same sex opponent / sexual displays	ad	M +<br>F +	[5]
Japanese quail<br>*Coturnix japonica*	T	Captive	Behavioural proactivity / Boldness	juv	M +<br>F +	[6]
Pied flycatcher<br>*Ficedula hypoleuca*	T & A4	Captive	Exploratory behaviour / Activity / Latency to resume activity after simulated predator attack	juv	M +<br>F =	[7]
Ring-necked pheasant<br>*Phasianus colchicus*	T	Captive	Spur length	ad	M – –<br>F –	[8]
Ring-necked pheasant<br>*Phasianus colchicus*	T	Captive	Digit ratio (2D:3D)	ad	M =<br>F +	[9]
Spotless starling<br>*Sturnus unicolor*	T & A4	Wild	Plasma androgen concentrations	juv	M +<br>F +	[10]
Zebra finch<br>*Taeniopygia guttata*	T	Captive	Habituation to novel food source / Latency to approach novel object	juv	M +<br>F + / =	[11]
Zebra finch<br>*Taeniopygia guttata*	T	Captive	Resting metabolic rate	juv	M +<br>F +	[12]

T: Testosterone; A4: androstenedione
Effects: + increased trait value; ++ strongly increased trait value; – reduced trait value; – – strongly reduced trait value; = trait not affected

References

1. Müller W, Groothuis TGG, Kasprzik A, Dijkstra C, Alatalo RV, et al. Prenatal androgen exposure modulates cellular and humoral immune function of black-headed gull chicks. Proc R Soc B. 2005;272: 1971-1977.

2. Pfannkuche KA, Gahr M, Weites IM, Riedstra B, Wolf C, et al. Examining a pathway for hormone mediated maternal effects - Yolk testosterone affects androgen receptor expression and endogenous testosterone production in young chicks (*Gallus gallus domesticus*). Gen Comp Endocrinol. 2011;172: 487-493.

3. Sandell MI, Tobler M, Hasselquist D. Yolk androgens and the development of avian immunity: an experiment in jackdaws (*Corvus monedula*). J Exp Biol. 2009;212: 815-822.

4. Strasser R, Schwabl H. Yolk testosterone organizes behavior and male plumage coloration in house sparrows (*Passer domesticus*). Behav Ecol Sociobiol. 2004;56: 491-497.

5. Partecke J, Schwabl H. Organizational effects of maternal testosterone on reproductive behavior of adult house sparrows. Dev Neurobiol. 2008;68: 1538-1548.

6. Daisley JN, Bromundt V, Mostl E, Kotrschal K. Enhanced yolk testosterone influences behavioral phenotype independent of sex in Japanese quail chicks *Coturnix japonica*. Horm Behav. 2005;47: 185-194.

7. Ruuskanen S, Laaksonen T. Yolk hormones have sex-specific long-term effects on behavior in the pied flycatcher (*Ficedula hypoleuca*). Horm Behav. 2010;57: 119-127.

8. Rubolini D, Romano M, Martinelli R, Leoni B, Saino N. Effects of prenatal yolk androgens on armaments and ornaments of the ring-necked pheasant. Behav Ecol Sociobiol. 2006;59: 549-560.

9. Romano M, Rubolini D, Martinelli R, Alquati AB, Saino N. Experimental manipulation of yolk testosterone affects digit length ratios in the ring-necked pheasant (*Phasianus colchicus*). Horm Behav. 2005;48: 342-346.

10. Müller W, Deptuch K, Lopez-Rull I, Gil D. Elevated yolk androgen levels benefit offspring development in a between-clutch context. Behav Ecol. 2007;18: 929-936.
11. Tobler M, Sandell MI. Yolk testosterone modulates persistence of neophobic responses in adult zebra finches, *Taeniopygia guttata*. Horm Behav. 2007;52: 640-645.
12. Tobler M, Nilsson JA, Nilsson JF. Costly steroids: egg testosterone modulates nestling metabolic rate in the zebra finch. Biol Lett. 2007;3: 408-410.
